# The SGLT-2 Inhibitor Dapagliflozin Has a Therapeutic Effect on Atherosclerosis in Diabetic ApoE^−/−^ Mice

**DOI:** 10.1155/2016/6305735

**Published:** 2016-12-26

**Authors:** Weiling Leng, Xinshou Ouyang, Xiaotian Lei, Mingxia Wu, Liu Chen, Qinan Wu, Wuquan Deng, Ziwen Liang

**Affiliations:** ^1^Department of Endocrinology, The First Affiliated Hospital of Third Military Medical University, Chongqing 400038, China; ^2^Department of Internal Medicine, Section of Digestive Diseases, Yale University of Medicine, New Haven, CT 06520, USA

## Abstract

*Background*. Our study aimed to observe the effect of sodium glucose cotransporter-2 (SGLT2) inhibitor dapagliflozin on diabetic atherosclerosis and investigate the subsequent mechanism.* Methods*. Aortic atherosclerosis was induced in streptozotocin induced diabetic ApoE^−/−^ mice by feeding with high-fat diet, and dapagliflozin was administrated intragastrically for 12 weeks as treatment. Effects of dapagliflozin on indices of glucose and fat metabolism, IL-1*β*, IL-18, NLRP3 protein levels, and the reactive oxygen species (ROS) were measured. The atherosclerosis was evaluated by oil red O and hematoxylin-eosin staining. The effects of dapagliflozin on the IL-1*β* production in culturing primary macrophages of wild type and NLRP3^−/−^ knockout mice were investigated for mechanism analyses.* Results*. Dapagliflozin treatment showed favorable effects on glucose and fat metabolism, partially reversed the formation of atherosclerosis, inhibited macrophage infiltration, and enhanced the stability of lesion. Also, reduced production of IL-1*β*, IL-18, NLRP3 protein, and mitochondrial ROS in the aortic tissues was detected with dapagliflozin treatment. In vitro, NLRP3 inflammasome was activated by hyperglucose and hyperlipid through ROS pathway.* Conclusions*. Dapagliflozin may be of therapeutic potential for diabetic atherosclerosis induced by high-fat diet, and these benefits may depend on the inhibitory effect on the secretion of IL-1*β* by macrophages via the ROS-NLRP3-caspase-1 pathway.

## 1. Introduction

Previous evidence from epidemiological studies has indicated that cardiovascular diseases (CVDs) have become the most serious complications of diabetes mellitus (DM), which is the leading cause of mortality and morbidity for DM patients. It has been well understood that pathophysiological changes in patients with DM, mainly the toxicity of high blood glucose to the endothelium and other cells of the vessels, may lead to the pathogenesis of atherosclerosis and subsequent CVDs. Although the key pathophysiological mechanisms underlying the so-called diabetic atherosclerosis were not fully determined, chronic inflammatory response, and associated lipid deposition, macrophage infiltration and smooth muscle cell proliferation in DM have been suggested to play important roles [1–3].

Recently, interleukin-1*β* (IL-1*β*) and interleukin-18 (IL-18) have been recognized as two inflammatory cytokines which contribute to the development of diabetic atherosclerosis [4], and the underlying mechanisms downstream of these cytokines have been evaluated. The inherent immunity has been linked to the activation of inflammatory response of the body. The inherent immunity recognizes damage-associated molecular patterns (DAMPs) and pathogen-associated molecular patterns (PAMPs) via pattern recognition receptors (PRRs) and thus excites the inflammatory reaction and starts the defense mechanism [5]. NLRP3, a NOD-like receptor and also an intracellular PRR, has been suggested to play a key role during the above pathophysiological process. The NLRP3 can be activated by several different exogenous and endogenous stimulation signals to form a multiprotein complex known as NLRP3 inflammasome. Previous studies indicated that ligand-binding NLRP3 can promote the formation of inflammasome and activate caspase-1, eventually facilitating the maturation and secretion of pro-IL-1*β* and pro-IL-18 [6, 7]. Recent studies have confirmed that NLRP3 inflammasome is involved in inflammatory response during the pathogenesis of atherosclerosis. Indeed, activation of NLRP3 inflammasome in macrophages has been involved in the cholesterol crystals formation in the pathogenesis of atherosclerosis [8]. Moreover, silence of NLRP3 gene has been found to delay the progression of atherosclerosis in mice [9]. Although IL-1*β* [10, 11] and IL-18 [12] have been suggested to be proatherosclerosis, whether they functions via regulation of NLRP3 inflammasome remains to be determined.

Sodium glucose cotransporters-2 (SGLT-2) inhibitors are a new class of antidiabetic medications targeting against renal glucose reabsorption. Dapagliflozin, as a SGLT-2 inhibitor, was marketed in 2012. The glucose lowering effect of dapagliflozin has been confirmed in many randomized controlled clinical trials which showed distinguished lowering effects of dapagliflozin for glycosylated hemoglobin (HbA1c), fasting blood glucose, and postprandial blood glucose [13, 14]. Moreover, besides the antidiabetic effect, dapagliflozin also seemed to be cardioprotective, by lowering blood pressure [15], reducing body weight [16], triglyceride and uric acid [17, 18], and improving insulin resistance [19]. Recently, a few studies indicated that SGLT-2 inhibitors may exert their cardiometabolic benefits via anti-inflammatory effects [20, 21]. However, the overall effects of dapagliflozin on atherosclerosis in DM and the potential benefits involved, for example, their effects on IL-1*β* and IL-18 cytokines and NLRP3 inflammasome systems, have not been evaluated. Randomized controlled clinical trials have proven that dapagliflozin can be used alone to treat the patients with type 2 diabetes mellitus and it has good effects in decreasing glycosylated hemoglobin (HbA1c), fasting blood glucose, and postprandial blood glucose [13, 14]. Interestingly, dapagliflozin can improve cardiovascular diseases in diabetes mellitus by slightly lowering blood pressure [15], reducing body weight [16], triglyceride and uric acid [17, 18], and improving insulin resistance [19]. In addition, there are trials showing that SGLT-2 inhibitors can reduce inflammatory markers in the serum and organs and thus decrease the degree of arteriosclerosis in the diabetic animal models [20, 21]. However, the effects of SGLT-2 inhibitors on inflammatory markers, including NLRP3 inflammasome and IL-1*β*, IL-18 cytokines have not been completely clarified yet. It is unclear that whether SGLT-2 inhibitors can affect atherosclerosis progression or not. Accumulated knowledge about effect of dapagliflozin on atherosclerosis and its underlying mechanisms can bring us more information about its safety and practicability in clinic.

The aims of the present study were to explore the effects of dapagliflozin on aortic atherosclerosis in diabetic versus nondiabetic ApoE^−/−^ mice and detect the possible underlying mechanism, particularly their influence on the ROS-NLRP3-caspase-1 pathway.

## 2. Methods

### 2.1. Mice

The spontaneous atherosclerotic lesions in apolipoprotein E knockout (ApoE^−/−^) mice after the hypercholesterolemia have been widely used as a mouse model of atherosclerosis in previous studies [22]. Consistently, we used this atherosclerotic mouse model in our study. Briefly, 40 male ApoE^−/−^ mice (6 weeks old, Beijing HFK Bioscience Co., Ltd.) and the 6 male C57BL/6J mice (6 weeks old, The Experimental Animal Center of the Third Military Medical University) were all bred in the animal rooms of the First Affiliated Hospital of the Third Military Medical University. The specific conditions were as follows: a SPF environment, temperature (18–26°C), relative humidity (55%), and 12 h light/12 h dark cycle. The animal cages and drinking bottles were disinfected regularly, the beddings were sterilized in an autoclave, and the animal rooms were disinfected with ultraviolet lamps periodically. After one-week accommodation, the ApoE^−/−^ mice were fed with a high-fat diet (HFD, general diet 79.85% + fat (oil) 15% + custard powder 5% + cholesterol 0.15%) to induce atherosclerosis of the aorta, while the C57BL/6J mice were fed with a general diet. The animal diets were purchased from the Experimental Animal Center of the Third Affiliated Hospital of the Third Military Medical University. Male ApoE^−/−^ mice (12 weeks old, 20 to 24 g) were intraperitoneally injected STZ (Sigma-Aldrich, St. Louis, MO) at the dose of 130 mg/kg dissolved in 100 mM citrate buffer (pH4.5) to induce DM, while the controls received buffered saline alone. After one week, we measured the blood glucose levels of all mice using a glucometer (Abbott Diabetes Care Inc. Optium Xceed) by tail vein puncture blood sampling. After 4 weeks, the mice with blood glucose > 16.7 mmol/l served as diabetic group according to the previous studies [23], and the non-STZ-injected ApoE^−/−^ mice served as nondiabetic group. The diabetic and nondiabetic mice were randomly assigned to treatment group (*n* = 12) and control group (*n* = 8) separately. The mice in treatment group received intragastrically dapagliflozin (AstraZeneca) 1.0 mg/kg/d for 12-week treatment, while C57BL/6J mice and mice in control group were intragastrically given vehicle 1.0 mg/kg/d. All animals received human care and all study protocols were approved by the Institutional Animal Care and Use Committee of the Third Military Medical University before performance.

### 2.2. Metabolic Measurement

After intraperitoneal injection of STZ, the blood glucose and body weight of mice were measured weekly. At an age of 28 weeks, all mice were subject to 8–12 h fasting and then anesthetized by intraperitoneal injection of 10% chloral hydrate. Thereafter, the blood was collected via orbital vein and then centrifuged (4°C, 3000 r × 15 min) to separate the serum. A part of serum was used to detect the serum concentration of total cholesterol (TCH), triglyceride (TG), high density lipoprotein cholesterol (HDL-c), low density lipoprotein cholesterol (LDL-c), and free fatty acid (FFA) at Clinical Laboratory of the First Affiliated Hospital of the Third Military Medical University, and the rest were preserved at −80°C for the measurements of the serum levels of NLRP3, IL-1*β*, and IL-18 with ELISA kits (Shanghai Jinglai Biotech, shanghai, China) according to the methods and procedures provided by the reagent manufacturer.

### 2.3. Quantification of Atherosclerotic Lesion Area

After blood collection via orbital vein, the aorta of the mice was dissected with fat removed under a surgery microscope. The aortic root was embedded with OCT and then made into 5 *μ*m frozen sections. Thereafter, the sections were dried, soaked in 60% isopropanol for 10 min, stained with oil red O (Sigma-Aldrich, O0625, USA) working fluid (3 : 2) for 10 min, differentiated in isopropanol for 3–10 s till the interstitium was clear, and then washed with water. The obtained sections were counter-stained with Mayer hematoxylin, differentiated in 1% Hcl-alcohol for 30 s, blued with running tap water, carefully dried with filter papers, and mounted with glycogelatin. The upper segments of aortas were fixed in 4% paraformaldehyde solution, embedded in paraffin, and sectioned at 5 *μ*m. After dehydration, sections were stained with hematoxylin-eosin (HE, Beyotime, Shanghai, China). After removal of adjacent fat, the upper segments of thoracic aorta and abdominal aorta were flatly placed on the slides, differentiated in 60% isopropanol for 10 min, stained in oil red O working fluid (3 : 2) for 3 h, differentiated in 60% isopropanol for 6-7 times till the background color became white, and then photographed. The area of lesion in the sections of aorta and aortic root was calculated using Image-pro plus 6.0 (MediaCybernetics, Inc.).

### 2.4. Immunofluorescent Staining

The frozen sections of aortic root were fixed with ice-acetone for 10 min, added with 0.3% Triton X-100 to make cell membrane permeabilized, and blocked with 5% BSA at room temperature for 1 h. Then they were blocked at 4°C overnight with primary antibodies (MOMA-2 antibody 1 : 50, Abcam; *α*-SMA antibody 1 : 400, Sigma), and incubated at room temperature for 3 h using secondary antibodies (1 : 200 and 1 : 400) protecting from light for coloration by antigen-antibody binding. Finally they were counterstained for nuclei with DAPI and mounted with the fluorescer. Macrophages and smooth muscle actin were observed under confocal fluorescence microscope (Zeiss LSM 780) and the positively stained macrophages and smooth muscle cells were automatically analyzed and quantified on Image-pro plus 6.0.

### 2.5. ROS Assay

To investigate aortic atherosclerotic lesions ROS levels, isolated arteries were loaded with DHE (1 *μ*mol/L) for 30 minutes at 37°C. The ROS were observed under confocal fluorescence microscope (Zeiss LSM 780), and DHE turn red fluorescent upon oxidation.

### 2.6. Isolation and Culture Macrophages from Bone Marrow

WT and NLRP3^−/−^ KO male mice bone marrow-derived macrophages (BMDMs) were obtained by differentiating bone marrow. Briefly, bone marrow cells were flushed out of femurs and tibias and the remaining cells were maintained in different medium. BMDMs were maintained in macrophage differentiating medium (DMEM and M-CSF (40 ng/mL, Sigma)) for 5 days. The medium was supplemented with 10% FBS (Hyclone Laboratories), 2 mM L-glutamine, penicillin (100 U/mL), and streptomycin (100 *μ*g/mL). To test the direct action of dapagliflozin on macrophages, the WT macrophages were plated into the sterile 96-well plate, stimulated with LPS (100 ng/mL, Sigma) for 3 h, and then, respectively, cultured with 0.2 M palmitate (PA, Sigma), high glucose (HG, 33 mmol/L), and PA+HG DMEM medium for 24 h. Finally the medium was added with dapagliflozin (12.5 *μ*M) and new-prepared ATP (5 mM) for 30 min. Then we collected the supernatant of cell culture to assay the content of IL-1*β* with ELISA kits (Shanghai Jinglai Biotech, shanghai, China). To test the action pathway of PA or HG on IL-1*β* production, the WT and NLRP3^−/−^ KO macrophages were plated into 6-well plate, stimulated with LPS for 3 h, and then, respectively, cultured with 0.2 M PA, HG (33 mmol/L), and PA+HG DMEM medium for 24 h and collected cell lysates to test NLRP3 and caspase-1 expression with Western blot.

### 2.7. Western Blot

The total aorta tissue was homogenized. The proteins of tissues and cell lysates were then separated by SDS-PAGE and further blotted using following specific antibodies: (anti-NLRP3 antibody 1 : 1000, anti-ASC antibody 1 : 1000, anti-caspase-1 antibody 1 : 400, anti-IL-1*β* antibody 1 : 1000, and anti-IL-18 antibody 1 : 500) (Novus biological, Littleton, Co., USA). The membranes were scanned with Typhoon (Pharmacia, USA) and quantitated using Quality One. The experiments were repeated for 3 times.

### 2.8. Statistical Analysis

All data were presented as means ± standard deviation (M ± SD). The nonparametric rank sum test or analysis of variance (ANOVA) was applied to evaluate the differences among the groups. A *p* < 0.05 indicates a statistically significant difference. All statistical analyses were performed on SPSS (20.0, Inc., Chicago, IL, USA).

## 3. Results

### 3.1. Changes of Metabolic Parameters after Dapagliflozin Treatment

**Figure 1:**
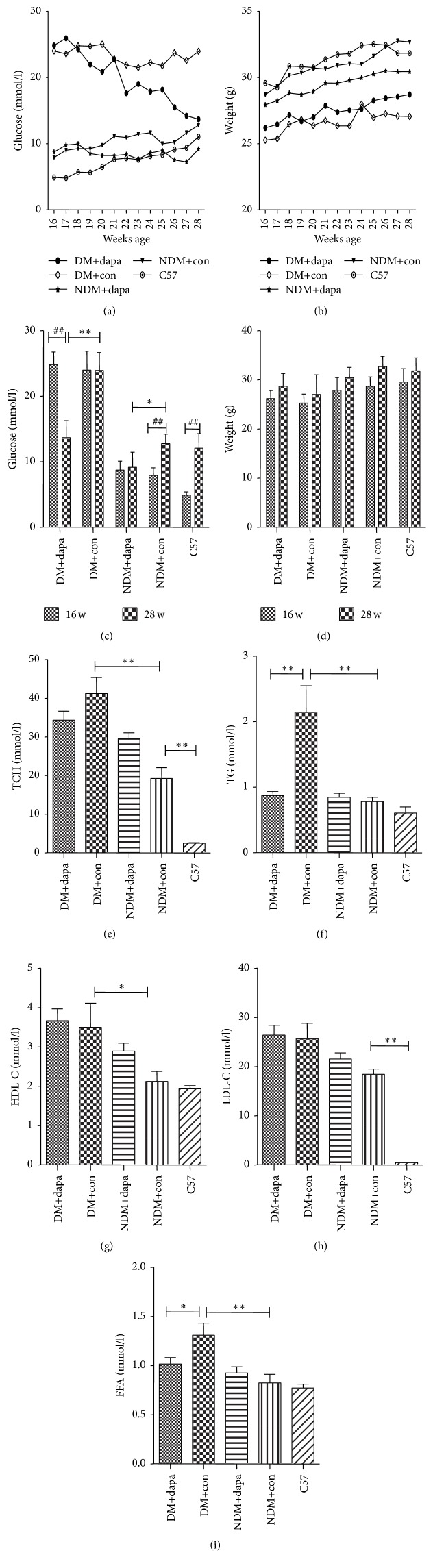
Changes of metabolic parameters in mice from each group. (a) and (c) Compared with non-DM group, the blood glucose in DM group was significantly increased; and dapagliflozin treatment of 12 wks significantly decreased blood glucose in both groups; (b) and (d) no significant changes of body weight were detected before and after dapagliflozin treatment in each group; (e), (g), and (h) the levels of cholesterol were significantly increased in the ApoE^−/−^ mice fed with a high-fat diet, particularly in DM ApoE^−/−^ mice. Dapagliflozin treatment did not reduce the levels of TCH, HDL-c, or LDL-c; (f) and (i): dapagliflozin treatment decreased the levels of TG and FFA. *∗*, *p* < 0.05; *∗∗*, *p* < 0.01; ##, *p* < 0.01.

The fasting body weight, blood glucose, and blood lipid levels of mice in each group measured during the study were shown in Table 1. No significant changes of body weight were detected in DM or non-DM ApoE^−/−^ mice that received dapagliflozin. However, the levels of blood glucose, TCH, TG, and FFA of diabetic ApoE^−/−^ mice were markedly increased as compared with nondiabetic ApoE^−/−^ mice. More importantly, after treatment with dapagliflozin, the fasting blood glucose decreased by 43% (*p* < 0.01) in the DM ApoE^−/−^ mice (*p* < 0.05), while HDL-c, TC, and LDL-c levels were merely affected (Figure 1).

### 3.2. Changes of Serum NLRP3 Inflammasome, IL-1*β*, and IL-18 after Dapagliflozin Treatment

**Figure 2:**
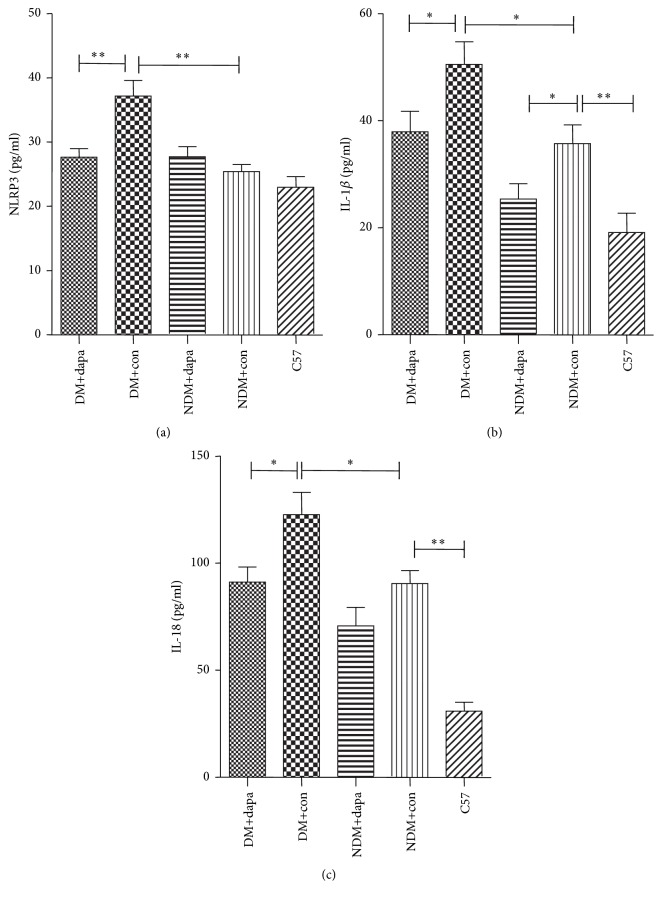
Changes of serum NLRP3 inflammasome, IL-1*β*, and IL-18 levels in mice from each group. (a), (b), and (c) represented the serum levels of NLRP3, IL-1*β*, and IL-18, respectively. After 12 weeks of treatment with dapagliflozin, the serum levels of NLRP3, IL-1*β*, and IL-18 significantly reduced in DM mice. ^*∗*^*p* < 0.05; ^*∗∗*^*p* < 0.01.

As shown in Table 2, compared with those of the non-DM mice, DM mice had significantly increased serum levels of NLRP3 (*p* < 0.01), IL-1*β* (*p* < 0.05), and IL-18 (*p* < 0.05). Moreover, serum IL-1*β* and IL-18 levels in nondiabetic control group were also significantly increased as compared with those of the C57BL/6J group (*p* < 0.01). More importantly, after 12-week treatment with dapagliflozin in DM mice, the serum levels of NLRP3 (*p* < 0.01), IL-1*β* (*p* < 0.05), and IL-18 (*p* < 0.05) proteins were all significantly reduced, while NLRP3 and IL-18 levels were not significantly affected (Figure 2).

### 3.3. Dapagliflozin Attenuated Atherosclerotic Lesion Formation in ApoE^−/−^ Mice

**Figure 3:**
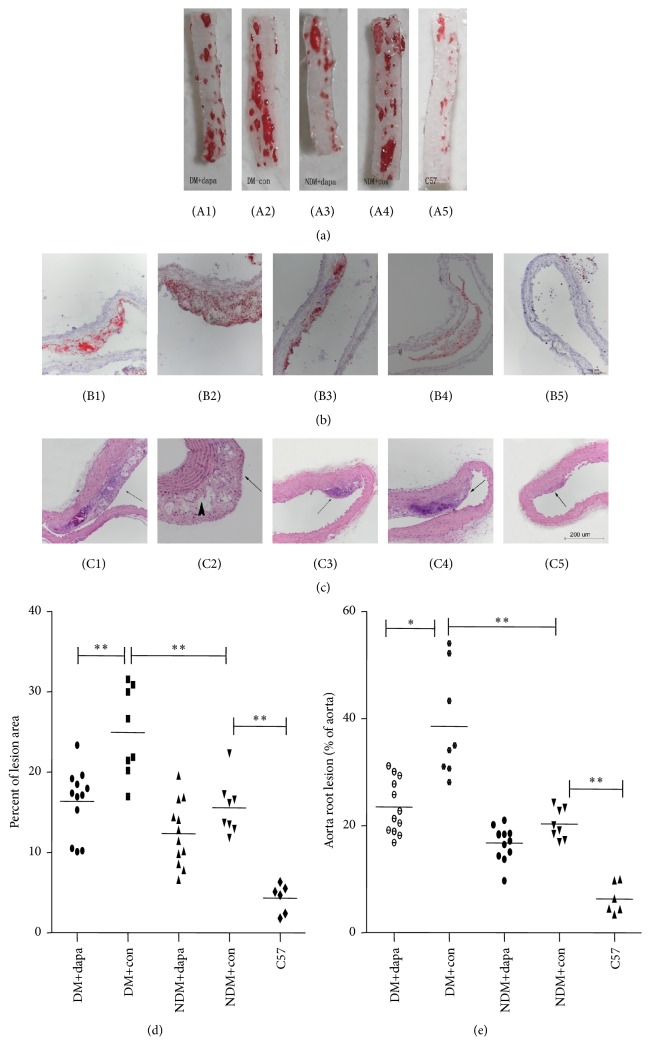
Dapagliflozin attenuated the formation of atherosclerotic lesion in ApoE^−/−^ mice. (a) Oil red O staining of thoracic aorta and abdominal aorta. Numbers 1–5 indicated diabetic treatment group, diabetic control group, nondiabetic treatment group, nondiabetic control group, and C57BL/6J control group, respectively; (b) oil red O staining of aortic roots (100x); (c) HE staining of aortic roots (100x), the small black arrow showed lesions and the big black arrow showed the necrotic lipid core of lesions; (d) percentage of arterial lesions in aorta; (e) percentage of lesions at aortic root in lumen cross-sectional area; ^*∗*^*p* < 0.05; ^*∗∗*^*p* < 0.01.

The pathological images of the aorta stained with oil red O of the mice from each group were shown in Figure 3(a) 1–5. Atherosclerotic lesions of STZ-induced DM mice were 60% larger as compared with those of non-DM ApoE^−/−^ mice (*p* < 0.01). Compared with normal C57BL/6J mice, non-DM ApoE^−/−^ mice had a 262% increment in atherosclerotic lesions (*p* < 0.01). Importantly, dapagliflozin treatment was associated with reduced area of aortic lesions in DM mice by 34% (*p* < 0.01). Moreover, dapagliflozin treatment also slightly reduced the formation of aortic lesions in non-DM group. Similarly, pathological analyses of the aortic roots (Figure 3(b)) also showed that dapagliflozin treatment decreased the area of lesion by 64% (*p* < 0.01) in DM mice and by 21% (*p* < 0.05) in non-DM mice as compared with the controls. HE staining of paraffin sections showed that the aortic walls of normal C57BL/6J mice were uniform in thickness and a few atherosclerotic lesions were observed. In other four groups, aortic walls were accompanied by a series of changes, including thickened arterial endothelium and aortic wall to different extents, newly formed fibrous cap, and overt atherosclerotic lesions. The formation of cholesterol crystals was found in diabetic group, and dapagliflozin treatment significantly reduced the cholesterol crystals in diabetic treatment group (Figure 3(c)). Taking together, these results supported that dapagliflozin treatment confers antiatherosclerotic effects in DM mice.

### 3.4. Dapagliflozin Altered the Content of Macrophages and SMCs in Atherosclerotic Lesions

**Figure 4:**
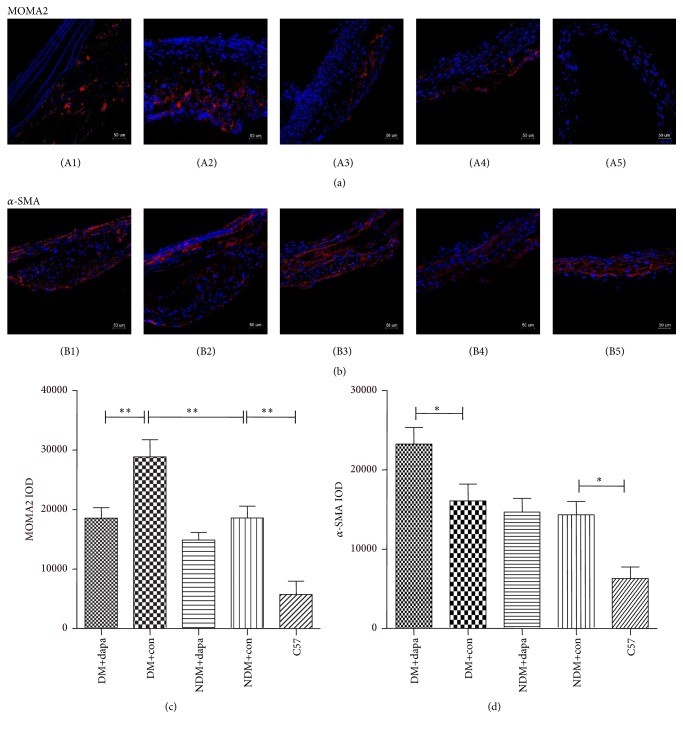
Influence of dapagliflozin on cellular formation (macrophage infiltration and smooth muscle cells) in atherosclerotic lesion in the aorta. Numbers 1–5 indicated diabetic treatment group, diabetic control group, nondiabetic treatment group, nondiabetic control group, and C57BL/6J control group, respectively. (a) Macrophage infiltration in atherosclerotic lesions (red: macrophages; blue: nuclei); (b) smooth muscle cell infiltration in atherosclerotic lesions (red: *α*-SMA antibody labeled smooth muscle cells; blue: DAPI-labeled nuclei); (c) immunofluorescence optimal density of MOMA2 antibody; (d) immunofluorescence optimal density of *α*-SMA antibody. ^*∗*^*p* < 0.05; ^*∗∗*^*p* < 0.01.

Staining of aortic tissues with specific markers of MOMA-2 and *α*-SMA further determined the effects of dapagliflozin on the cellular formation of atherosclerotic lesions in DM mice. We found that the increased macrophage infiltration in atherosclerotic lesions was reversed after dapagliflozin in DM ApoE^−/−^ mice (Figure 4). Moreover, dapagliflozin treatment also restored the reduced content of smooth muscle cells in lesions in the DM ApoE^−/−^ mice (Figure 4). These results may highlight that dapagliflozin may improve the vulnerability of the atherosclerotic lesions by regulation the cellular formation of the lesion in DM ApoE^−/−^ mice.

### 3.5. Effect of Dapagliflozin on ROS Production and Inflammatory Cytokine Levels in Aortic Atherosclerotic Tissues

**Figure 5:**
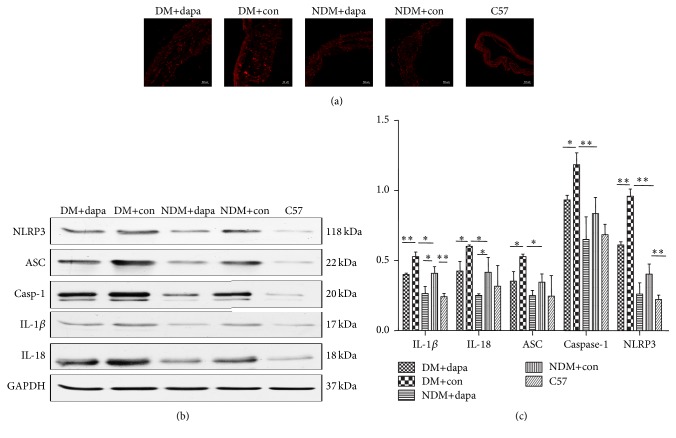
Dapagliflozin attenuated the formation of ROS and NLRP3 inflammasome in DM ApoE^−/−^ mice. Decreased ROS formation in aortic tissues of diabetic ApoE^−/−^ mice was attenuated by dapagliflozin treatment (a). The expression of NLRP3 inflammasome, IL-1*β*, and IL-18 in aortic tissues of mice from each group (b). Western blot analysis was used to detect the protein expression levels with the antibodies against NLRP3, ASC, caspase-1, IL-1*β*, and IL-18. GAPDH was used as loading control (c). ^*∗*^*p* < 0.05; ^*∗∗*^*p* < 0.01.

We conducted ROS measurements to determine the effects of dapagliflozin on the generation of ROS in aortic atherosclerotic lesions with DHE and found that diabetic ApoE^−/−^ mice demonstrated an elevation in vascular ROS production and attenuated by dapagliflozin treatment (Figure 5(a)). Compared with non-DM control group, DM mice were associated with increased NLRP3 (*p* < 0.01), ASC (*p* < 0.05), caspase-1 (*p* < 0.01), IL-1*β* (*p* < 0.05), and IL-18 (*p* < 0.05) proteins in abdominal aorta, while dapagliflozin treatment partly inhibited the expressions of the above proteins in aorta tissues (Figure 5(b)). These results suggested that dapagliflozin can reduce the ROS production and NLRP3 inflammasome activation in aortic root of the DM mice.

### 3.6. Effect of Dapagliflozin on NLRP3 Inflammasome In Vitro

**Figure 6:**
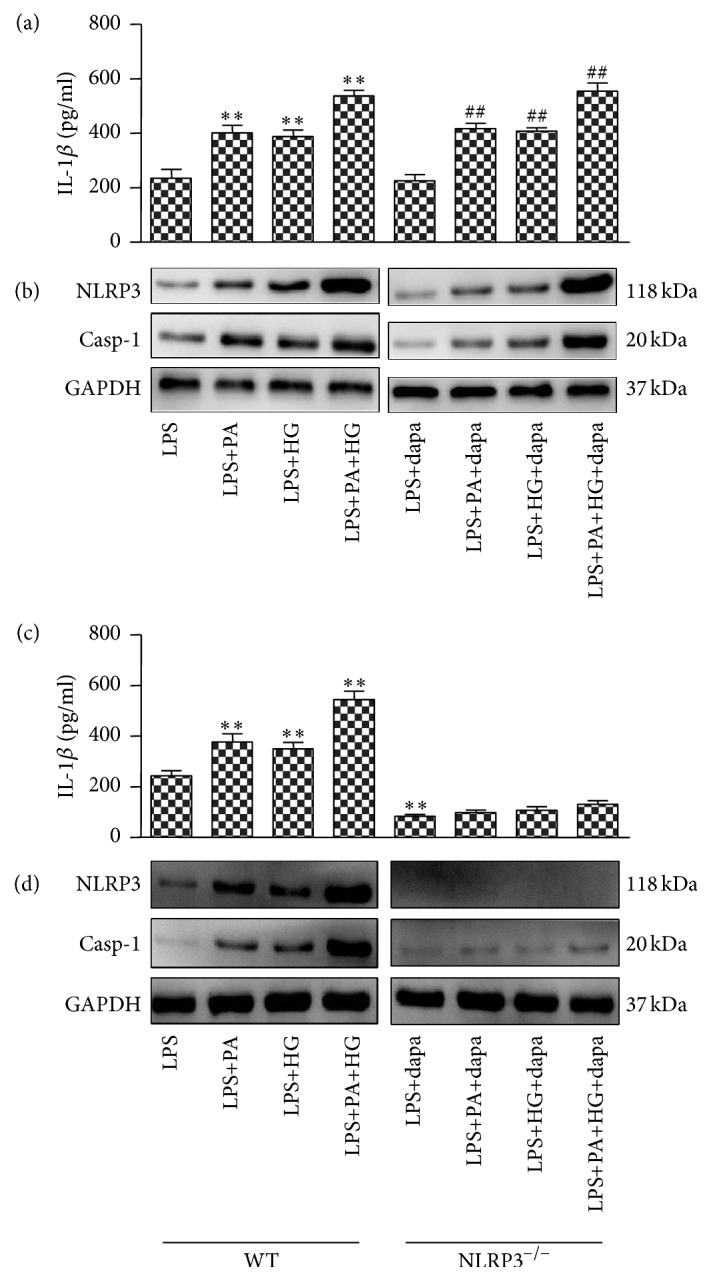
Effect of dapagliflozin on the macrophages was investigated with different stimulations. BMDMs from WT were treated with LPS, LPS+PA, LPS+HG, or LPS+PA+HG for 24 h and then with dapagliflozin and ATP for 30 m and tested the level of IL-1*β* in the supernatant (a). Collecting the cell lysates and testing the expression of NLRP3 and caspase-1 with Western blot. Data show mean ± SD; *n* = 3. ^*∗*^*p* < 0.05; ^*∗∗*^*p* < 0.01 versus LPS. ^##^*p* < 0.01 versus LPS+dapa (b). The WT and NLRP3^−/−^ mice macrophages were isolated, cultured, and stimulated with LPS, LPS+PA, LPS+HG, or LPS+PA+HG for 24 h and then assayed the IL-1*β* in supernatant (c) and detected the expression of NLRP3 and caspase-1 in cell lysates with Western blot. Data show mean ± SD; *n* = 3. ^*∗*^*p* < 0.05; ^*∗∗*^*p* < 0.01 versus WT+LPS (d).

To investigate the direct effect of dapagliflozin on macrophage, the WT primary macrophages were treated LPS and then PA or HG and finally incubated with dapagliflozin (12.5 *μ*M) for 30 m. Dapagliflozin could not directly activate or inhibit the NLRP3 inflammasome activation in macrophages (Figures 6(a) and 6(b)). PA and HG stimulation increased the expression of NLRP3 and production of IL-1*β* in WT macrophages, special combining together. However, in NLRP^−/−^ macrophages, the stimulation action of LPS + PA + HG on the expression of NLRP3 and production of IL-1*β* almost disappeared (Figures 6(c) and 6(d)).

## 4. Discussion

Previous findings from a number of clinical studies have indicated that some antidiabetics and lipid-lowering medications (e.g., pioglitazone, statins, and fibrates) were associated with reduced cardiovascular events in patients with type 2 DM [24–26]. Dapagliflozin is known as a glucose lowering medication which functions via inhibition of SGLT-2, thus inhibiting renal glucose reabsorption. However, the cardiometabolic influence of dapagliflozin and potential mechanisms underlying its benefits to attenuation of atherosclerosis in DM has not been comprehensively evaluated. In this study, by using a model of high-fat diet STZ treated ApoE−/− mice, we found that dapagliflozin treatment was associated with significant improvement in indices of lipids and glucose metabolism. Moreover, dapagliflozin partially reversed the formation of aortic atherosclerotic lesions in DM mice, and these benefits may involve the inhibition of NLRP3 inflammasome. Our results highlighted the benefits of dapagliflozin for attenuating of diabetic atherosclerosis and provide further evidence for its application in DM patients.

We found that dapagliflozin had significant glucose lowering effect in DM mice, and this was consistent with findings of previous studies [13, 14]. Interestingly, it also decreased the blood glucose of non-DM ApoE^−/−^ mice. After treatment for 12 weeks, dapagliflozin significantly lowered TG and FFA levels, but not TCH, HDL-C, and LDL-C in DM ApoE^−/−^ mice, suggesting that dapagliflozin had favorable lipid regulation effect in DM ApoE^−/−^ mice. A study by Terasaki et al. [27] also evaluated the effects of dapagliflozin on blood lipids, although the results of the study which showed that dapagliflozin did not significantly affect TG or FFA was different from ours. This could be explained by the different duration of dapagliflozin treatment between the 2 studies, and four-week treatment with dapagliflozin was too short to reduce the TG and FFA levels in diabetic mice. FFA plays an important role in stimulating NLRP3 inflammasome activation and IL-1*β* and IL-18 inflammation cytokines releasing [28].

Our subsequent pathologic analysis revealed that dapagliflozin remarkably inhibited the formation and development of aortic lesions in diabetic ApoE^−/−^ mice, which indicates that dapagliflozin could delay the formation of diabetic atherosclerosis. The underlying mechanisms of the benefits of dapagliflozin on diabetic atherosclerosis may be multiple, and chronic improvement of glucose and lipids metabolisms may play important roles. Besides, inhibition of inflammatory response during the pathogenesis of atherosclerosis may also be involved. Chronic inflammation response plays a key role in the diabetic atherosclerosis and macrophages have been recognized as the principal inflammation reaction cells. On one hand, macrophages can be transformed into foam cells and the necrotic foam cells can form a lipid core which is the main component of atheromatous lesions; on the other hand, macrophages are the major part of cell composition in atheromatous lesions, and several cytokines secreted by the cells could change the local environment and cause the instability of lesions and the development of disease [1, 29]. The smooth muscle cells (SMCs) migrating into the lesions are capable of producing collagen fibers which are the main source of fibrous cap in the lesions. Reduced SMCs in the fibrous cap or increment of macrophages can make fibrous cap thin and vulnerable to rupture [30]. Therefore, suppressing the macrophage infiltration in the lesions as well as preventing the reduction of SMCs has been considered as the treatment targets to reduce the vulnerability of lesion rupture. In our study, dapagliflozin was found to prevent the infiltration of macrophages in the arterial lesions of DM mice and prevent the loss of SMCs in the lesions, which suggest its benefit for lesion stability.

In the local chronic inflammation response of atheromatous lesions, NLRP3 inflammasome was activated via ROS production, which was related to the release of IL-1*β* and IL-18 from macrophages. Our results showed that DM mice had elevated serum NLRP3, IL-1*β*, and IL-18 proteins as compared with the nondiabetic mice, which might be related to the high glucose and FFA state. Zhou et al. proposed that hyperglycemic exposure was a key inducer for the production of IL-1*β* and the activation of NLRP3 inflammasome in pancreas [31]. Wen et al. found that the increased FFAs induced by a high-fat diet could activate NLRP3 inflammasome in macrophages [32]. Our study found that dapagliflozin could decrease the serum levels of NLRP3, IL-1*β*, and IL-18 proteins in diabetic ApoE^−/−^ mice and IL-1*β* level in nondiabetic ApoE^−/−^ mice, indicating that the benefits of dapagliflozin in diabetic atherosclerosis may involve both the systematic and regional anti-inflammatory effects, perhaps by inhibition of NLRP3 inflammasome system. Moreover, dapagliflozin inhibited the expression of NLRP3, ASC, caspase-1, IL-1*β*, and IL-18 in DM mice, and such expression was also decreased in the nondiabetic atherosclerosis model (not statistically significant).

**Figure 7:**
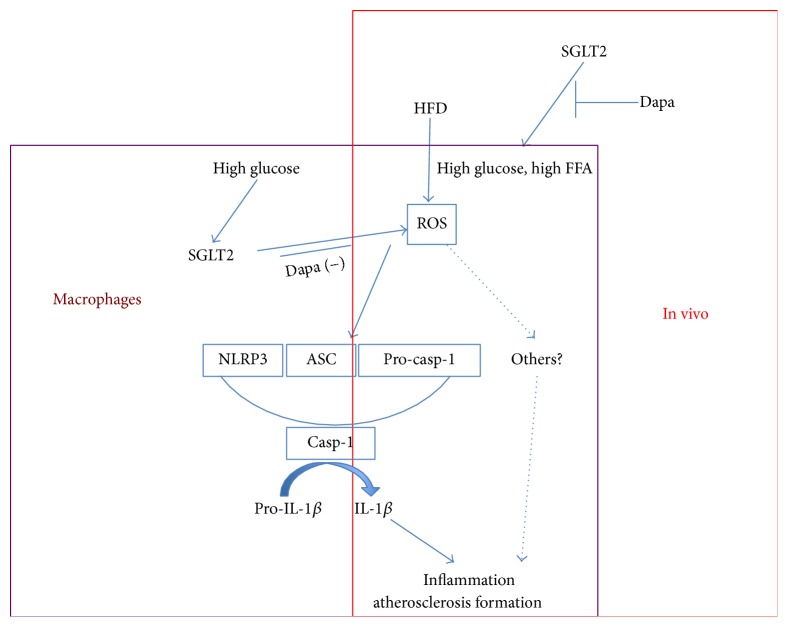
The possible signal pathway of high glucose and high FFA activation NLRP3 inflammasome through ROS production and the possible interfering site of dapagliflozin.

As we all know, three models for activation of the NLRP3 inflammasome have been proposed: the channel model, the lysosome rupture model, and the ROS model [33]. All NLRP3 agonists trigger the generation of ROS, which activates the NLRP3 inflammasome via the ROS-sensitive thioredoxin-interacting (TXNIP) protein. The important contributions of oxidative stress to atherosclerosis have been well established; for example, Di Marco et al. demonstrated that diabetes-accelerated atherosclerosis is associated with elevated ROS [34]. So we further tested the level of ROS in aortic atherosclerotic lesions. Our results found that diabetes- accelerated atherosclerosis is associated with an increase in IL-1*β* and IL-18 via the activation of NLRP3 inflammation by a process that involves the generation of ROS, and treatment with dapagliflozin prevented the diabetes-associated increase in IL-1*β* and IL-18 and ROS production and attenuated the accelerated development of atherosclerosis in the aortic root. We also investigated the action of dapagliflozin on NLRP3 inflammasome in culturing primary macrophages. PA or HG caused activation of the NLRP3 inflammasome and triggered the generation of IL-1*β* in WT macrophages, but the action was canceled in NLRP3^−/−^ macrophages. Dapagliflozin had not direct action on macrophages. Our results show that dapagliflozin may be inhibit ROS-NLRP3 pathway by means of reducing blood sugar and lipid and then reduce the production of IL-1*β* and IL-18. To the best of our knowledge, this study is the first to elucidate that dapagliflozin treatment was associated with inhibition of the secretion of IL-1*β* by macrophage via the ROS-NLRP3-caspase-1 pathway in diabetic atherosclerosis (Figure 7). The potential molecular signaling underling the inhibitory effect of dapagliflozin on NLRP3 inflammasome system deserves further evaluation.

## 5. Conclusions

Results of our study indicated that SGLT2 inhibitors dapagliflozin can attenuate the formation of atherosclerotic lesion and increase the stability of lesion by inhibiting the NLRP3 inflammasome in diabetic atherosclerosis, which provide further evidence for its benefits in DM patients

## Figures and Tables

**Table 1:** Data on body weight, fasting blood glucose, and blood lipid profile in all groups.

	DM+dapa	DM+con	NDM+dapa	NDM+con	C57
BW (g)	28.71 ± 2.58	27.05 ± 3.97	30.44 ± 2.06	32.68 ± 2.12	31.83 ± 2.66
FBG (mmol/l)	13.69 ± 2.58^##^	23.94 ± 2.73	9.17 ± 2.28^*∗*^	12.79 ± 1.42	12.05 ± 2.24
TC (mmol/l)	34.34 ± 7.97	41.26 ± 11.75	29.56 ± 5.28	19.29 ± 7.84	2.48 ± 0.41^&&^
TG (mmol/l)	0.87 ± 0.22^##^	2.14 ± 1.15	0.85 ± 0.21	0.78 ± 0.19	0.61 ± 0.23
HDL-c (mmol/l)	3.67 ± 1.05	3.50 ± 1.73	2.89 ± 0.70	2.12 ± 0.74	1.93 ± 0.19
LDL-c (mmol/l)	26.42 ± 6.75	25.71 ± 8.85	21.56 ± 4.06	18.43 ± 3.06	0.45 ± 0.14^&&^
FFA (mmol/l)	1.02 ± 0.22^#^	1.31 ± 0.34	0.92 ± 0.23	0.83 ± 0.24	0.77 ± 0.10

Diabetic mice that received dapagliflozin (DM+dapa), diabetic mice that received vehicle (DM+con), nondiabetic mice that received dapagliflozin (NDM+dapa), nondiabetic mice that received vehicle (NDM+con), and wild-type (C57/BL6) mice were measured. BW: body weight; FBG: fasting blood glucose; TC: total cholesterol; TG: triglycerides; HDL-c: HDL cholesterol; LDL-c: LDL cholesterol; FFA: free fat acid; values are expressed as mean ± SD, per group. #, *p* < 0.05; ##, *p* < 0.01 versus DM+con; *∗*, *p* < 0.05 versus NDM+con; &&, *p* < 0.01 versus NDM+con.

**Table 2:** The concentration of serum NLRP3, IL-1*β*, and IL-18 in each group.

	DM+dapa	DM+con	NDM+dapa	NDM+con	C57
NLRP3 (pg/ml)	27.69 ± 4.46^##^	37.17 ± 6.94	27.71 ± 5.37	25.43 ± 3.10	22.98 ± 4.04
IL-1*β* (pg/ml)	37.91 ± 13.32^#^	50.51 ± 12.05	25.38 ± 9.95^*∗*^	35.73 ± 9.93	19.10 ± 8.91^&&^
IL-18 (pg/ml)	91.15 ± 24.90^#^	122.67 ± 29.71	70.85 ± 29.74	90.54 ± 18.10	30.95 ± 10.08^&&^

The concentrations of serum NLRP3, IL-1*β*, and IL-18 were measured. Values are expressed as mean ± SD. #, *p* < 0.05; ##, *p* < 0.01 versus DM+con; *∗*, *p* < 0.05 versus NDM+con; &&, *p* < 0.01 versus NDM+con.
